# Experimental investigation on the physical, microstructural, and mechanical properties of hemp limecrete

**DOI:** 10.1038/s41598-023-48144-y

**Published:** 2023-12-19

**Authors:** Siva Avudaiappan, Pablo Ignacio Cuello Moreno, Luis Felipe Montoya R, Manuel Chávez-Delgado, Krishna Prakash Arunachalam, Pablo Guindos, Teresita Marzialetti B, Pablo Fernando Parra, Erick I. Saavedra Flores, Julio Ignacio Flores Arrey

**Affiliations:** 1 Departamento de Ciencias de la Construcción, Facultad de Ciencias de la Construcción y Ordenamiento Territorial, Universidad Tecnológica Metropolitana, Santiago, Chile; 2 Department of Chemical Engineering, Faculty of Engineering, University of Concepción, Concepción, Chile; 3 Departamento de Ingeniería Civil, Facultad de Ingeniería, Universidad Andres Bello, 4300866 Sede Concepción, Chile; 4 Department of Civil Engineering, University College of Engineering Nagercoil, Anna University, Nagercoil, India; 5 Department of Structural and Geotechnical Engineering, Pontificia Universidad Católica de Chile, Av. Vicuña Mackena, 7820436 Santiago, Chile; 6 Centro Nacional de Excelencia para la Industria de la Madera (CENAMAD) , Pontificia Universidad Católica de Chile, Av. Vicuña Mackenna, 4860 Santiago, Chile; 7 Faculty of Engineering and Sciences, Universidad Adolfo Ibáñez, 7941169 Santiago, Chile; 8 Department of Civil Engineering, Faculty of Engineering, University of Santiago of Chile, Santiago, Chile

**Keywords:** Civil engineering, Mechanical properties

## Abstract

This paper investigates the hemp limecrete mechanical and microstructural performance of a new sustainable and environmental friendly building material. Several studies have investigated the hemp limecrete focusing on the non-structural applications. The newly developed hemp limecrete consists of high mechanical and microstructural properties. The specimens were prepared with varying lengths and proportions of hemp fibers with lime and tested for compressive strength, flexural strength, thermal conductivity and microstructural analysis like SEM and EDS. The study found that the optimal fiber content for making mortars was between 2 and 4%. This conclusion was reached after analyzing the influence of fiber length and ratio on the properties of the mortars. The dry unit weight decreased when the fiber content was higher than 4%. In terms of strength, the study found that the flexural strength of the hemp limecrete improved with an increase in fiber ratio, but the compressive strength decreased. However, with 2% hemp fiber, compressive strengths of 3.48 MPa and above were obtained. The study also highlighted the good thermal insulation properties and dimensional stability of hemp limecrete. These findings have important implications for the use of hemp limecrete as a sustainable building material. The results suggest that hemp limecrete has the potential to be a viable alternative to conventional concrete in specific applications, particularly in areas where environmental sustainability is a priority.

## Introduction

It would be beneficial to establish connections between the agricultural sector and the building industry in order to design housing that is both sustainable and cheap in the future. Building can only be sustainable if it employs the utilization of renewable resources or materials reclaimed from construction debris^1^. In the current article, we looked at hemp's (Cannabis sativa) potential as a construction material when combined with hydraulic lime. Hemp is a rapid-growing evergreen perennial plant that may reach a height of 1.5 m to 4 m when cultivated^2^. About 40% of all energy consumed and 36% emissions of co_2_ are caused by the built environment^3^. Consequently, it is crucial to create carbon-negative, environmentally friendly building materials with minimal embodied energy to substitute cement-based technologies. Concrete made from hemp and lime is referred to as hemp–limecrete. The binder in this concrete is lime In the 1980s and early 1990s in France, it replaced daub and wattle to lighten Portland cement (PC) concrete used to restore historic buildings. Since then, hundreds of buildings throughout Europe have been built using or retrofitted with this ecologically friendly material.

The carbon negative nature of the lime concrete is one of its most remarkable eco-benefits. When lime is manufactured, carbon dioxide (CO_2_) is released into the atmosphere; however, this is balanced out by the hemp crop's which has the ability to store carbon. Hemp takes in carbon dioxide (CO_2_) as it grows, and this carbon is then incorporated into the plant's structure. As a result, the carbon does not escape from the walls made of hemp and lime. According to the findings of Boutin et al. (2006), the production of 1 m^2^ of hemp–lime wall with a thickness of 260 mm takes between 370 and 394 megajoules of energy and absorbs between 14 and 35 kg of carbon dioxide over the course of its lifetime^4^. In addition to having a strong reputation for being environmentally friendly nature, hemp limecrete also have a good performance on thermal conductivity. In addition to its eco-friendliness, hemp limecretes also have great insulating properties due to their high thermal capacity, medium density, and low thermal conductivity. The purpose of this study assesses the impact of the binder on the durability and mechanical strength (biodeterioration, salt exposure, and freeze–thaw resistance) of hemp limecrete. Hemp limecrete is made using hydraulic lime to accelerate the setting and hardening processes.

Since the hemp aggregate is capable of absorbing a significant amount of water i.e., in 24 h it can absorb 325% water of its own weight^5^, which has the potential to inhibit hydration, several of the concretes that were studied included a water retainer^6^. Hemp fibre is a excellent reinforcing material due to its high tensile strength and tolerance to alkaline environments, according to Li et al.^7^. This makes hemp fibre an ideal material for usage in reinforcing materials. When combined with a cementitious binder, hemp creates a building material that is distinct from conventional concrete in terms of its mechanical, thermal, and acoustic properties. This is because hemp fiber have a low density and a high porosity, meaning that there are a lot of spaces between the fiber. Because it has a superior capabilities on acoustic insulation, lower thermal conductivity and lower density, so it is be beneficial to employ in building^7–9^. Lime–hemp concrete (LHC) is a construction material that consists of hemp fiber, hydraulic lime, hydrated lime, water, and admixtures and may be used in conjunction with a load-bearing timber framework to create walls, etc. Its composition determines whether or not it may be utilized in floors or roofs^10^. These days, you can get a wide range of hemp and lime goods from a number of European firms that may be used to make an LHC. In addition to being able to support weight, a good wall should be able to meet the requirements for these other functions as well: insulation, soundproofing, airtightness, fireproofing, and protection against moisture. Traditional brick or wooden walls consist of many levels, each of which serves one or more of the aforementioned purposes. With the exception of a load-bearing hardwood structure and lime rendering, LHC walls don't need any other supplementary construction materials to operate properly^11^.

Hemp concrete is often used in conjunction with a load-bearing structure since it is not a load-bearing material individually. In spite of this, its mechanical strength is significant since it is the most often determined characteristic which permits comparison between the various binders. Due to its inherent weakness while wet, hemp concrete requires rapid development of its compressive strength (which could be greater than twice as substantial as its dry state). Compressive strengths for blends with a 2:1 ratio of (hemp by weight to binder) may vary anywhere from 0.12 to 0.2 MPa^9,11–15^, with the majority of the variance coming from factors such as density, binder type, and age. Previous studies^16–18^ have established that flexural strength is between 0.06 and 1.2 MPa, despite the fact that flexural strength is low overall. According to Elfordy et al.^19^ the disorderly organization and the hemp particles ductility nature are responsible for the poor strength of the hemp concrete. According to Nguyen, the concrete has a lesser compressive strength in comparison to other lightweight concretes^17^ because of the large porosity of the shiv.

There is a difference of view concerning the impact that the hydraulicity of the binder has in determining the strength of the concrete. It was discovered by Hirst et al. (2010) that the strength may not rise for the strength of the binder^14^. Nguyen (2010) claims that stronger binders increase strength if the hemp's capacity to absorb water does not limit its hydraulicity^17^. Murphy et al. 2010 found that concretes prepared using a hydraulic industrial binder had greater final flexural and compressive strengths than those made using lime, and that strength development relied on the binder's hydraulicity^13^. Additionally, De Bruijn et al. (2009) demonstrated that cement-rich binders may achieve greater compressive strengths^21^. There hasn't been a lot of investigation on how long hemp concrete will last. Lime, on the other hand, has been employed in construction projects since ancient times, and it is often combined with organic materials like wood (beams and lath), hair or straw. These kinds of buildings have shown to be very long-lasting. Homes constructed from hemp–lime has been in France for more than 20 years without any major weathering or durability issues being documented^22^. As a result of the lime's high alkalinity, the material has a reputation for being resilient to insects and mould, which could be supported by empirical evidence^23^.

Carbonation, alkali-aggregate interaction, chloride penetration, sulphate attack and freeze–thaw cycle are the primary issues in concrete durability. Biodeterioration, salt crystallization and freeze–thaw action were believed to be among the most potential instigators for the hemp concrete because of the absence of steel reinforcement, silica-based aggregate or tri-calcium aluminate. A mortar's freeze and thaw resistance mainly hinge on its pore structure's ability to endure strain from water freezing. According to a study of resistance to the freeze–thaw of lime and cement mortars conducted by Botas et al. the authors found that resistance to freeze–thaw reduced when lime hydraulicity is improved^24^, most likely as a result of the mortar's micropores. In addition, it was found that the air entraining agents had no effect on the mortar's resistance to freeze:thaw, while increasing its overall permeability. The influence of the hemp limecrete mortars' stronger mechanical strength overcomes the pore structural features, which indicates how they showed a greater resistance. A "wall combination" hemp limecrete was able to withstand severe freeze–thaw of 20 cycles, but it was discovered that limes that were either too hydraulic or inappropriate changed after just two cycles^25^. Additional study has shown that the compressive strength of concrete does not reduce even after being subjected to 25 freeze–thaw cycles^26^. Ilija Bošković^27^ study employs life cycle assessment to analyze greenhouse gas emissions from hemp–lime concrete. It reveals that variables such as hemp shiv sequestration, binder carbonation, and transport distances significantly impact emissions. Optimistic scenarios showed a negative global warming potential, while pessimistic ones were positive. The study also emphasizes the influence of hemp shiv degradability on emissions, highlighting the material's environmental sensitivity. Moletti et al.^28^ study examines the hygrothermal properties of hemp–lime, a sustainable building material made from lime-based binders and hemp shives. By conducting experiments over three years and using numerical simulations, the research demonstrates that the material's performance improves with maturation, primarily attributed to binder carbonation and reduced initial moisture content, as confirmed by XRD and TG-DTG analyses. Rotem et al.^29^ study evaluates the environmental impact of lime hemp concrete (LHC) with unfired binders as a lime replacement compared to standard LHC and conventional building materials. In an arid region, it reveals that LHC with unfired binders can reduce energy consumption and CO_2_ emissions by up to 90%, making it a highly sustainable construction option.

Several studies have been published on how hemp shive affects material qualities, was previously stated. Although this information is useful, it is incomplete since the studies cited above focus on a subset of the features of composites and a subset of the proportions of fiber based on cement. The impact of shive size on the composite's other characteristics, like porosity, density, microstructural analysis and thermal heat conductivity, has to be investigated further for lime hemp limecrete. In the present investigation, we examined these consequences for hemp limecrete. Basic features of the hemp limecrete were also examined, including compressive strength, flexural, thermal conductivity and water absorption to see how much impact fiber had on these characteristics of the hemp specimens.

This study introduces groundbreaking innovations in construction materials and sustainable building. It introduces hemp limecrete as an eco-friendly construction material, offering a sustainable alternative to traditional concrete. The study precisely identifies the optimal combination of fiber content (4%) and length (1 cm) for hemp limecrete, providing essential guidelines for effective usage. The manuscript employs a holistic approach by analyzing mechanical properties, thermal conductivity, and microstructure through SEM and EDS. Emphasizing sustainability, it highlights hemp limecrete as a promising eco-friendly material, aligning with global trends in sustainable construction. These innovations collectively position hemp limecrete as a revolutionary, environmentally conscious choice for the construction industry.

## Research objective

The research objective of hemp limecrete is to investigate and optimize the mechanical, and physical properties of the material, as well as its potential for use as a sustainable and eco-friendly construction material. This may include studying factors such as the optimal ratio of hemp to lime, the effect of different additives or binders. Ultimately, the goal is to develop a durable and cost-effective alternative to traditional building materials that has a lower environmental impact. Previous researches have been done on Hemp cement mortar, the novelty of the research lies with the utilization of lime and hemp alone in the preparation of mortar with hemp. The findings provide important information for architects, engineers, and builders on the mechanical properties of hemp limecrete, and the optimal fiber content and length required for desirable results. With further research and development, hemp limecrete could play an increasingly significant role in the construction industry as a sustainable and environmentally friendly alternative to conventional building materials.

## Materials and methods

**Figure 1:**
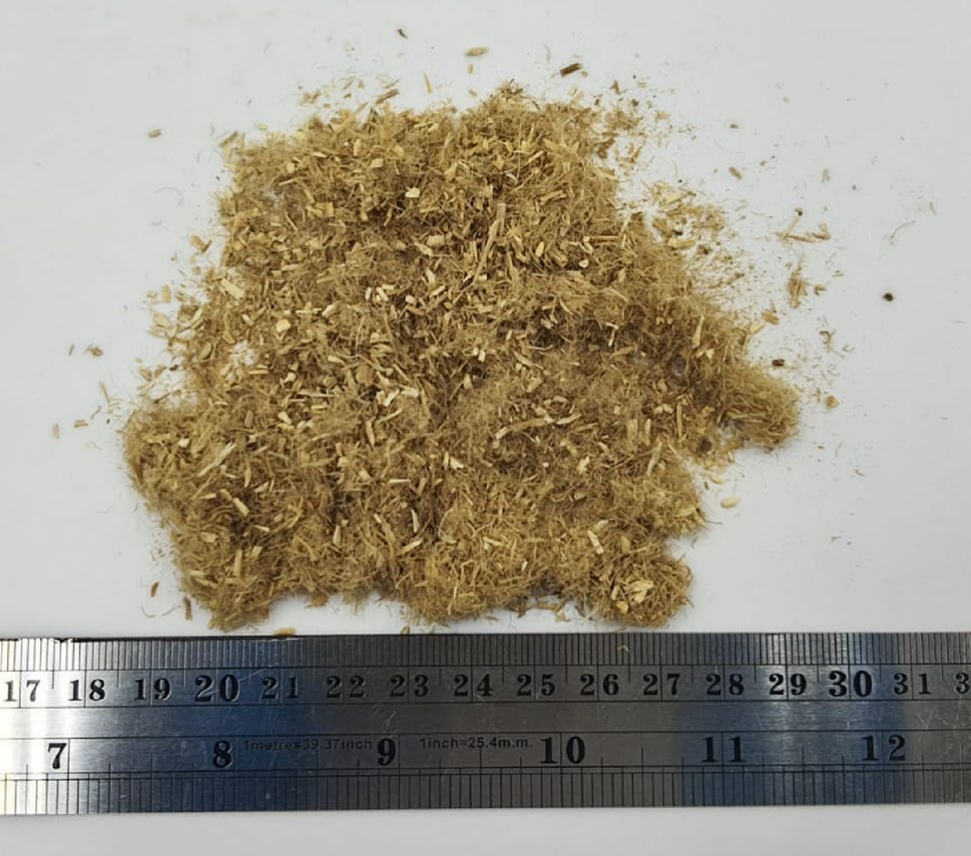
Hemp fiber.

The hemp came from the company Diamond Hemp Santiago, Chile. It was planted on April 12, 2021, and after drying out was harvested on May 3, 2022. All of the hemp plants were collected, packed, and stashed and the hemp hay were run through a mechanical shredder, and the resulting fiber, fibres, and dust were all utilized in this study. Hemp consisted of 67% fiber, 32% fibres and 3% dust (0.5 mm) by weight. Hemp Fiber used in this study is shown at Fig. 1 and Tables 1, 2 provides the physical and chemical properties of hemp fiber. Three samples of hemp were weighed and dried in a cabinet at 103 °C. for 24 h to assess the moisture content of the material. The hemp samples' moisture content on average is 13.3% after drying.

**Table 1:** Physical properties of hemp.

Specific gravity	Fiber length (cm)	Moisture absorption (%)		Tensile strength (MPa)	Elongation at break (%)
1.52	0.6–2.1		13	691	4

**Table 2:** Chemical properties of hemp.

Cellulose (%)	Lignin (%)	Hemicellulose (%)	Pectin (%)	Wax (%)
72–78	3.8–6.2	19–24	5–9	1.5

Hydraulic lime is used in this investigation, the chemical composition of lime from XRD analysis is shown in Table 3. The mixes were prepared using water-to-lime ratios of 0.35. We've tailored the quantity of hemp strands to the quantity of lime. There are 2%, 4%, and 6% of fibres by weight of the lime. Mixture ratios and approximate amounts are shown in Table 4. The mixture contains fibres of varying lengths and proportions. CS is the control Specimen and the mix properties were denoted as 2P0.5L, where P denotes the percentage of fibre and L denoted the length of fibre. There are ten distinct combinations here, nine of which include fibre and one of which is a control specimen. For the production of the cube specimens, we utilized industry-standard steel moulds with dimensions of the cylinder samples, 300 mm long, and breadth 150 mm. It was found that surrounding the mould using plastic sheet foil made it much simpler to remove the specimen once it had been prepared (O'Dowd & Quinn, 2005)^2^. The hemp was combined with 150 ml of water in a mixer for 5 min. At the same time, lime is combined together with water in a different container to produce a binder mixture. Hemp was then mixed with the binder mixture in the mixer for another five minutes. Any obvious lumps were manually broken apart. The mixture was poured into the moulds and compacted with a 45 mm wooden stave. Then a second layer was laid down and tamped, followed by a third. The mixture has been poured into the mould. The process for filling cylinders was quite similar. Tamping was followed by 1 min of vibration (at 50 Hz) in the moulds. After 24 h of curing in an indoor atmosphere of about 20 °C, the specimens were taken out of the moulds. After curing the specimens were subjected to mechanical strength tests. As a preliminary test, the mortar flow diameters were measured as per the ASTM C 1437 standard^30^. Vibrating mortars have been used to fill the moulds in two levels. The ASTM C 349^31^ and ASTM C 348^32^ standards have been followed to determine the compressive and flexural strengths after 7 and 28 days. Flexural strength test performed on 40 × 40 × 160 mm prism samples. After curing for 28 days, the samples were dried at 50 °C for 3 days, and the capillarity test was conducted. The quantity of water absorbed by capillary action was measured at 1, 5, 10, 20, 30, 60, 120, 180, 240, 300, 360, and 1440 min after water impermeability material was applied to the side surfaces of the samples. Archimedes' concept has been used to calculate the water absorption and porosity of the specimens.

**Table 3:** Chemical composition of lime in percentage.

CaO	SiO_2_	Fe_2_O_3_	K_2_O	Al_2_O_3_	SO_3_	MgO	P_2_O_5_	Cl	TiO_2_	Na_2_O	MuO	CaOCa_2_
84	1.2	14.1	7.57	7.7	3.65	1.7	2.5	1.95	0.68	0.52	0.13	1.16

**Table 4:** Mix ratios and quantities of materials.

Mix no	Fiber length (cm)	Fiber raito (%)	Lime	Water	Fiber
2P0.5L	0.5		400	150	4.5
2P1.0L	1.0	2	400	150	4.5
2P2.0L	2.0		400	150	4.5
4P0.5L	0.5		400	150	9.0
4P1.0L	1.0	4	400	150	9.0
4P2.0L	2.0		400	150	9.0
6P0.5L	0.5		400	150	13.5
6P1.0L	1.0	6	400	150	13.5
6P2.0L	2.0		400	150	13.5
CS	–	–	400	150	-

## Results and discussions

### Dry bulk density (BD) of hemp limecrete

**Figure 2:**
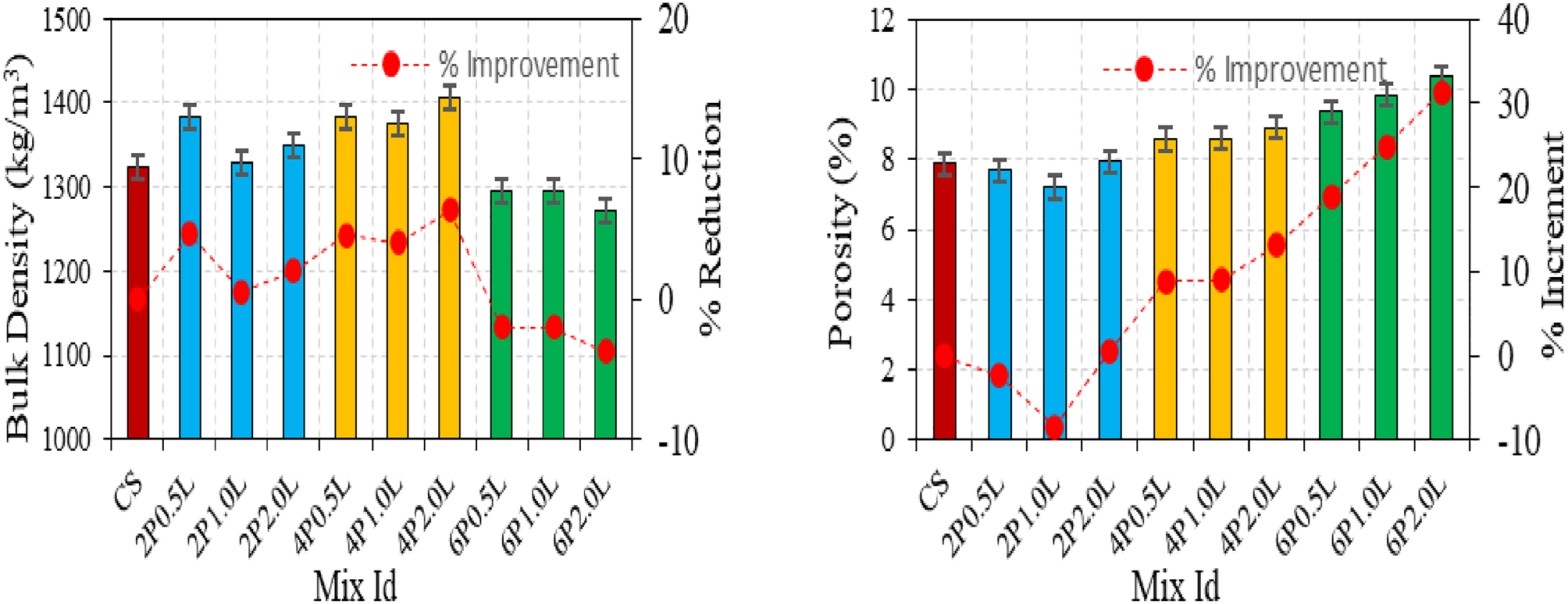
Results of bulk density and porosity of hemp limecrete.

**Figure 3:**
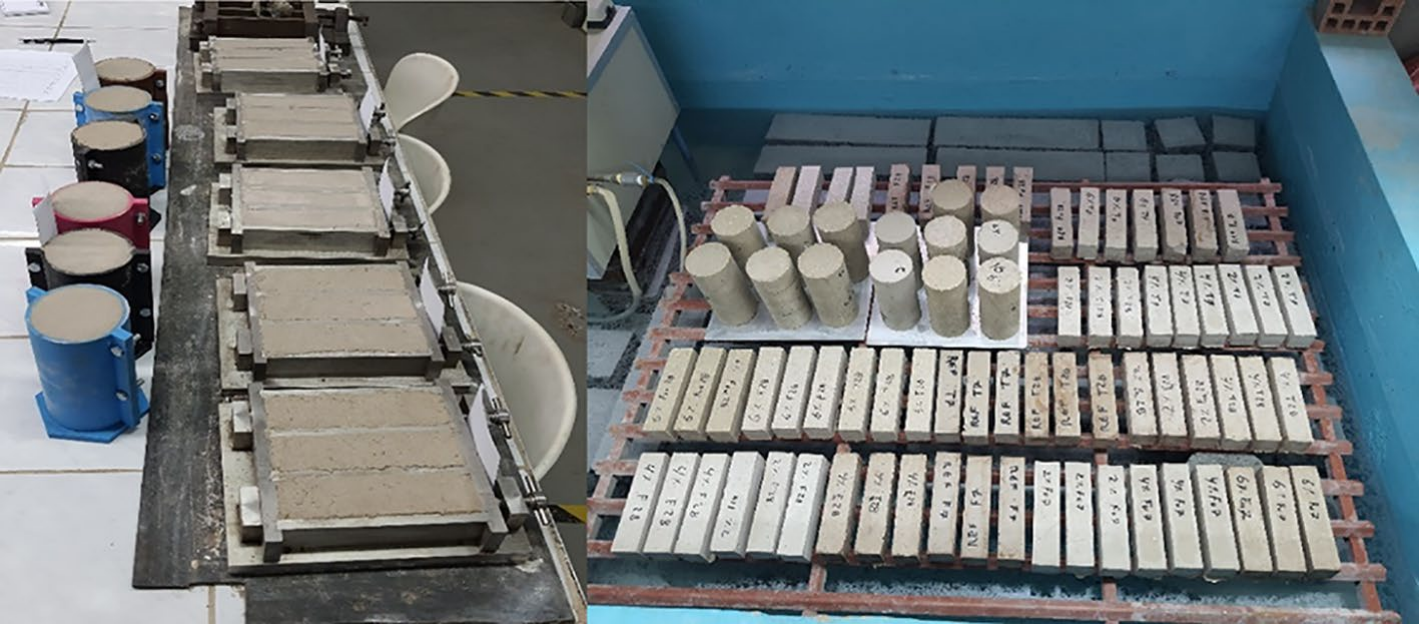
Casting and curing of specimens.

The Bulk density and porosity of hemp limecrete results were illustrated in Fig. 2. Bulk density values for fibrous mixtures can range anywhere from 1273 to 1407 kg/m^3^, on average. The Bulk density value of the fiber-free control mix is found to be 1323 kg/m^3^. The Bulk density of the mixes in which just 6% fibre included was found to be lower than that of the control mix, this is due to workability loss. When using 2 and 4% fibre, BD values are greater than the control specimen. Mortars' BD values increased with a rise in fibre ratio, as shown by a comparison of fibre ratios between 2 and 4%. The observed effects can be attributed to the fixed proportion of the mixing ratios, which are not volume-based. There has been a rise in the Bulk density values due to increase in the quantity of fibre. The addition of 6% fiber content made it challenging to place the mortars in the mold, which resulted in a hollow structure. Mortars with fiber lengths of 1 or 2 cm generally showed a decrease in bulk density values. On the other hand, using 4% fiber content and 2 cm long fibers led to BD values exceeding 1407 kg/m^3^. Mixtures containing 6% fiber showed Bulk density values below 1296 kg/m^3^. The decrease in processability caused by an increase in fiber length resulted in non-uniform distribution, which led to a general decrease in Bulk density values. Based on Fig. 3, it was observed that the porosity values of the fiber-based mixtures had a range of 7.21–10.35%. As the fiber ratio in the mixtures increased, the corresponding apparent porosity values also increased. Additionally, increasing the length of the fiber used further increased the apparent porosity values of the mixtures. Mixture compositions with 2% fiber content had porosity values that were closest to the control specimen porosity value of 7.72%. Interestingly, even the use of shorter fiber lengths, such as 0.5 cm and 1 cm, resulted in porosity values lower than the reference mixture's porosity value. Mixture compositions that utilized 0.5 cm long fibers exhibited greater homogeneity compared to other mixtures, as evidenced by the spread diameter of 14.4 cm. This increased homogeneity led to a reduction in porosity values. Some studies have suggested that short fibers act as micro aggregates and reduce porosity. The addition of 4% and 6% fiber results in porosity values is above 8%. The lowest BD value is achieved with a combination of 6% fiber and 2 cm fiber length, although the resulting porosity value is greater than 10%. Olivito and Zuccarello found in their study that increasing the length of steel fibers results in a minor decrease in unit volume weight values. The unit weight of mixtures was observed to increase as the steel fiber ratio increased, according to a study by Olivito and Zuccarello^33^. On the other hand, Asasutjarit et al. found in their study that an increase in fiber length led to a decrease in the density of composites and an increase in water absorption values^34^.

### Mechanical properties of mortars

**Figure 4:**
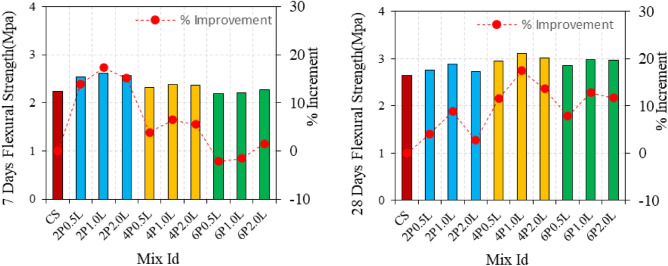
Flexure strength of hemp limecrete at 7 and 28 days.

**Figure 5:**
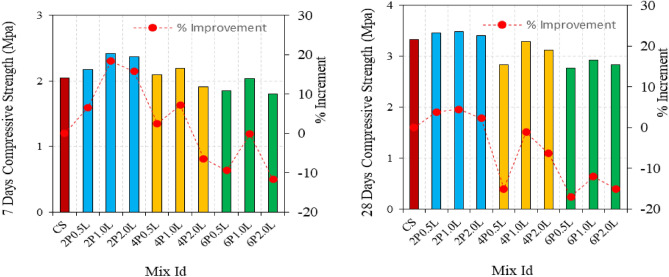
Flexure and compressive strength of hemp limecrete.

**Figure 6:**
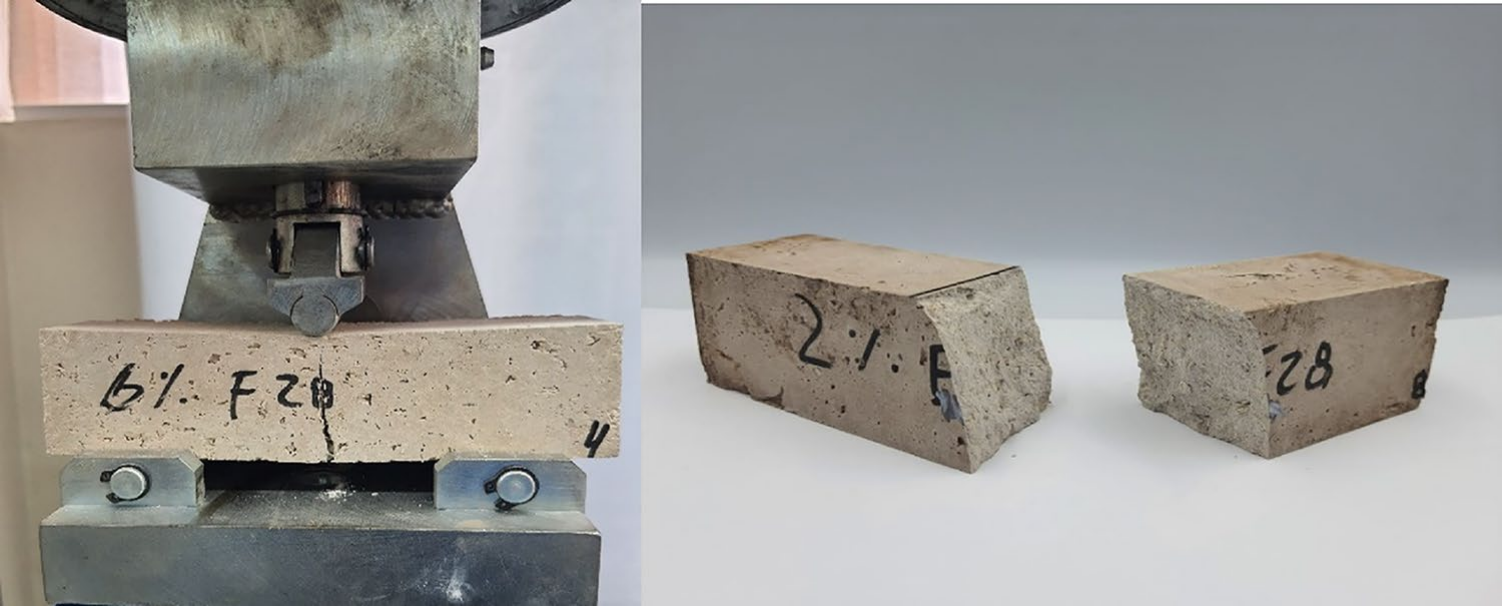
Flexural testing of hemp limecrete.

**Figure 7:**
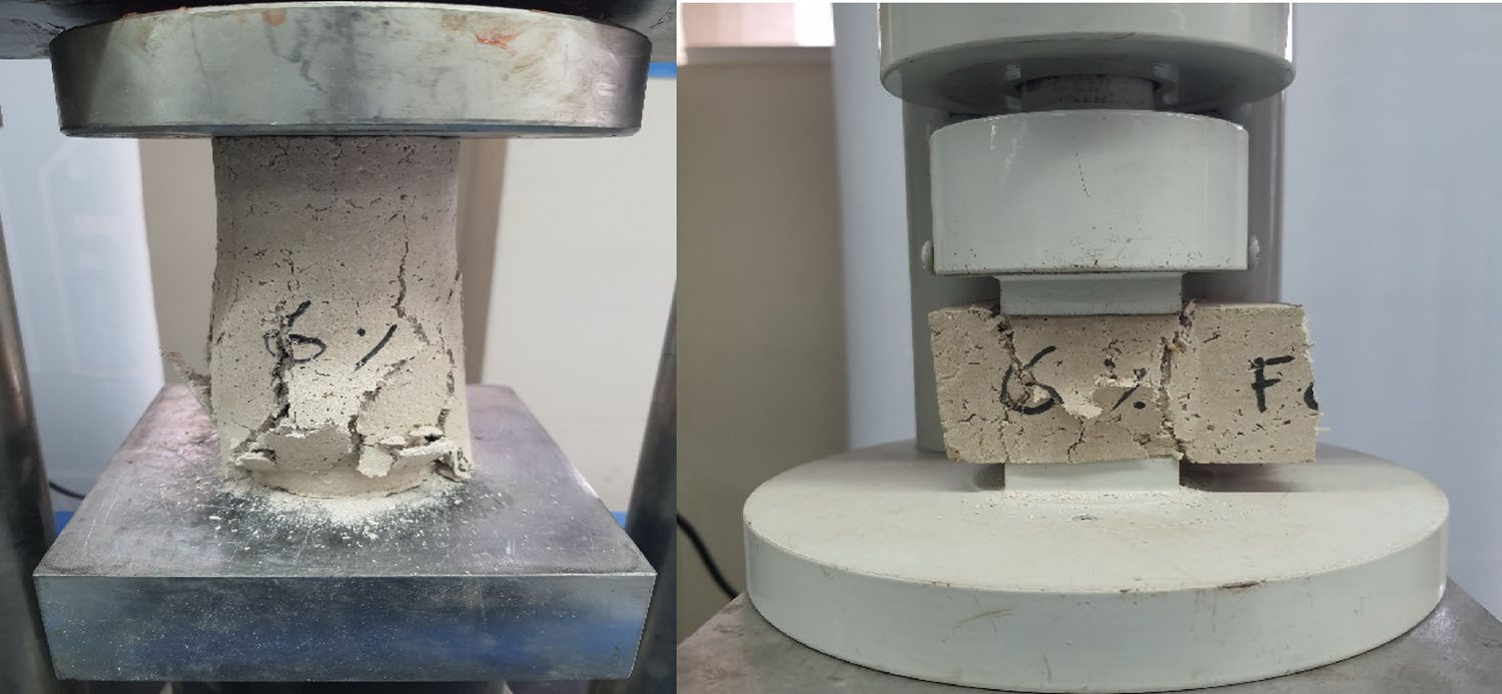
Compression testing of hemp limecrete.

Flexural strength and compressive strength outcomes of the hemp limecretes are shown in Figs. 4 and 5 for the 7 and 28-day. The flexure strength of the fiber-free mixture is 6.48 MPa. However, the 7 and 28-day flexural strength of the mortars do not show parallelism. The use of 6% 0.5 and 1 cm long fibers resulted in lower flexural strength than the reference mixture. Loss of workability have caused the reduction in flexural strength when using 0.5 and 1 cm fibers at 6% concentration. On the other hand, the use of 2 cm fibers increased the flexural strength, and generally, longer fibers resulted in higher flexural strength. The highest flexural strength was achieved with 2% fibers of 1 cm length, showing a 14.7% increase in strength compared to the control mix. However, increasing the fiber ratio caused a decrease in flexural strength. The flexural strength testing of the mortars are presented in Figs. 6 and 7. It is observed that the flexural strength of the mortars has significantly improved due to the increase in curing time. Unlike the 7-day strength results, an increase in the fiber ratio has led to an improvement in the 28-day flexural strength. However, the use of 6% fiber has still resulted in flexural strength values lower than 6.50 MPa. The 28-day flexural strength of all mixtures is higher than the reference mixture, with the highest strength achieved by the mixture containing 2 cm long fibers and 6% fiber content. The 7-day strength of this mixture was 6.58 MPa, which increased by 23.6% on the 28th day. The flexural strength generally increased with an increase in fiber length and ratio up to 4%, but reductions were observed at 6% fiber content. The optimal fiber length was found to be 1 cm with a 4% fiber ratio. The flexural strength of the fiber-matrix interface improved due to the increased curing time, particularly for natural fibers such as hemp, which may require longer curing periods. SEM analysis can help further explore these findings. Jonathan et al. observed an increase in flexure strength with an increase in hemp fiber ratio, while an increase in fiber length led to a decrease in flexure strength^35^.

The compressive strengths of the mixtures decreased with increasing fiber content, except for the mixture containing 2% 0.5 cm long fibers, which showed a 4% increase in compressive strength compared to the control specimen. It has also been noted that consistency of the mixture affects fiber orientation and ultimately, flexure strength^36^. The compressive strength of the mortars generally increases with the fiber length of 1 cm. Mixtures produced from 0.5 and 2 cm long fibers have similar properties. However, the compressive strength remains below the reference mixture when the fiber content is 6%. Figure 5 illustrates that the compressive strength increases with the increase in curing time. All blends produced with 2% fiber showed higher compressive strength than the reference blend. Nevertheless, the compressive strength of the mortars decreases as the fiber ratio increases. The compressive strength of mixtures with 6% fiber content is generally below 3 MPa, although a fiber length of 1 cm typically increases compressive strength. Similar results were observed in a study^14,37^. The use of 4% fiber content has resulted in an increase in flexure strength, despite the high porosity values of the mixtures. Mixtures with 4% and 6% fiber content exhibited decreased compressive strength due to high porosity. However, the fibers acted as reinforcement, preventing crack propagation and leading to an increase in flexure strength.

### Thermal conductivity on hemp mortar

**Figure 8:**
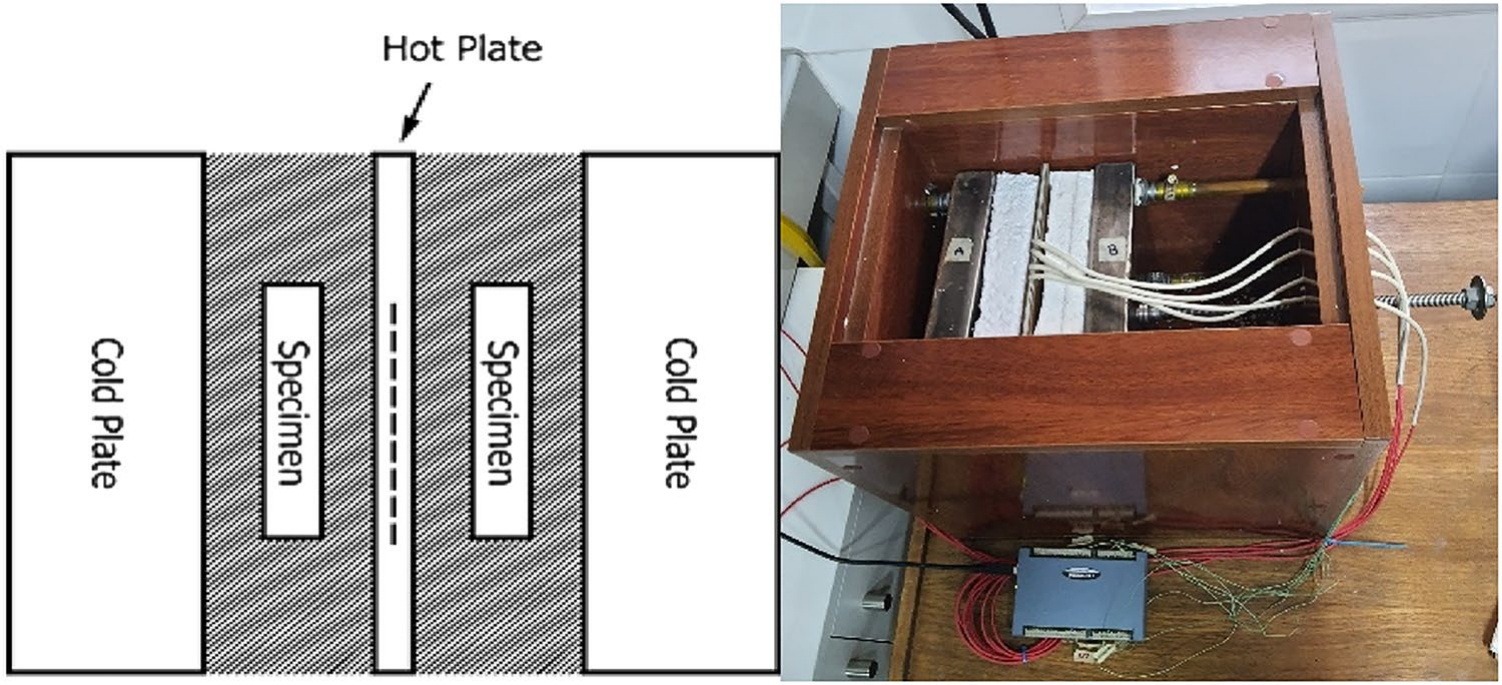
Thermal conductivity of hemp limecrete.

In order to determine the thermal conductivity of hemp limecrete, the study conducted thermal conductivity tests on three specimens of dimensions 160 mm × 40 mm × 40 mm. The tests were performed according to international standard ISO 8302^38^ utilizing the heat flow meter method with a plate apparatus as shown in Fig. 8. The testing specimens are dried to a consistent mass at 60 °C before the experiment. The test was carried out with a hot plate set at 25 °C, a cooling plate at 0 °C, and an average temperature of 12.5 °C was obtained. The accuracy of the absolute thermal conductivity measurement of the plate apparatus was determined to be within 2%. During the test, the heat moved through the sample in a path that was orthogonal to the direction in which the sample was being compacted. These tests provide important data for assessing the thermal insulation properties of hemp limecrete, which is a critical factor for its application as a sustainable building material.

The thermal conductivity of hemp limecrete with varying percentages of hemp was measured. The data shows that the thermal conductivity increases as the percentage of hemp increases, with the highest value of 0.27 W/mK obtained for the sample with 6% hemp. The sample with 4% hemp also showed a relatively high thermal conductivity of 0.23 W/mK. The samples with lower percentages of hemp, 2% and control specimen had lower thermal conductivity values of 0.19 W/mK and 0.17 W/mK, respectively. It is worth noting that the thermal conductivity values for the 2% and 6% hemp samples were quite similar, with only a small difference of 0.08 W/mK between them. These results suggest that adding hemp to lime can increase its thermal conductivity, which may have implications for its use in building insulation. However, it is also clear that the percentage of hemp used can have a significant impact on the thermal conductivity of the resulting composite, with higher percentages leading to higher values. The outcomes are consistent with those reported in the published research. Pavia and Walker^39^ also utilized the similar binder to fiber, but their composites have density ranges from 508 to 627 kg/m^3^. The results are line with evidence in the existing literature^40^. Using the same binder-to-fibers ratio as in the previous case, Walker and Pavia^39^ obtained composites with a higher density have a higher thermal conductivity ranging from 0.107 to 0.128 W/(m K)). In other studies^41^, composite fibers with an average length less than 4 mm had a higher thermal conductivity coefficient than those with an length greater than 4 mm, eventhough the difference was small as well as the thermal permittivity ebbed and flowed is around 0.12 W/(m K). In additional investigations^41^, the composite comprising fibers with an average length less than 2 mm had a greater thermal conductivity coefficient than those with an length of 2 mm average, the difference in thermal conductivity coefficients were similar and modest.

### SEM analysis

**Figure 9:**
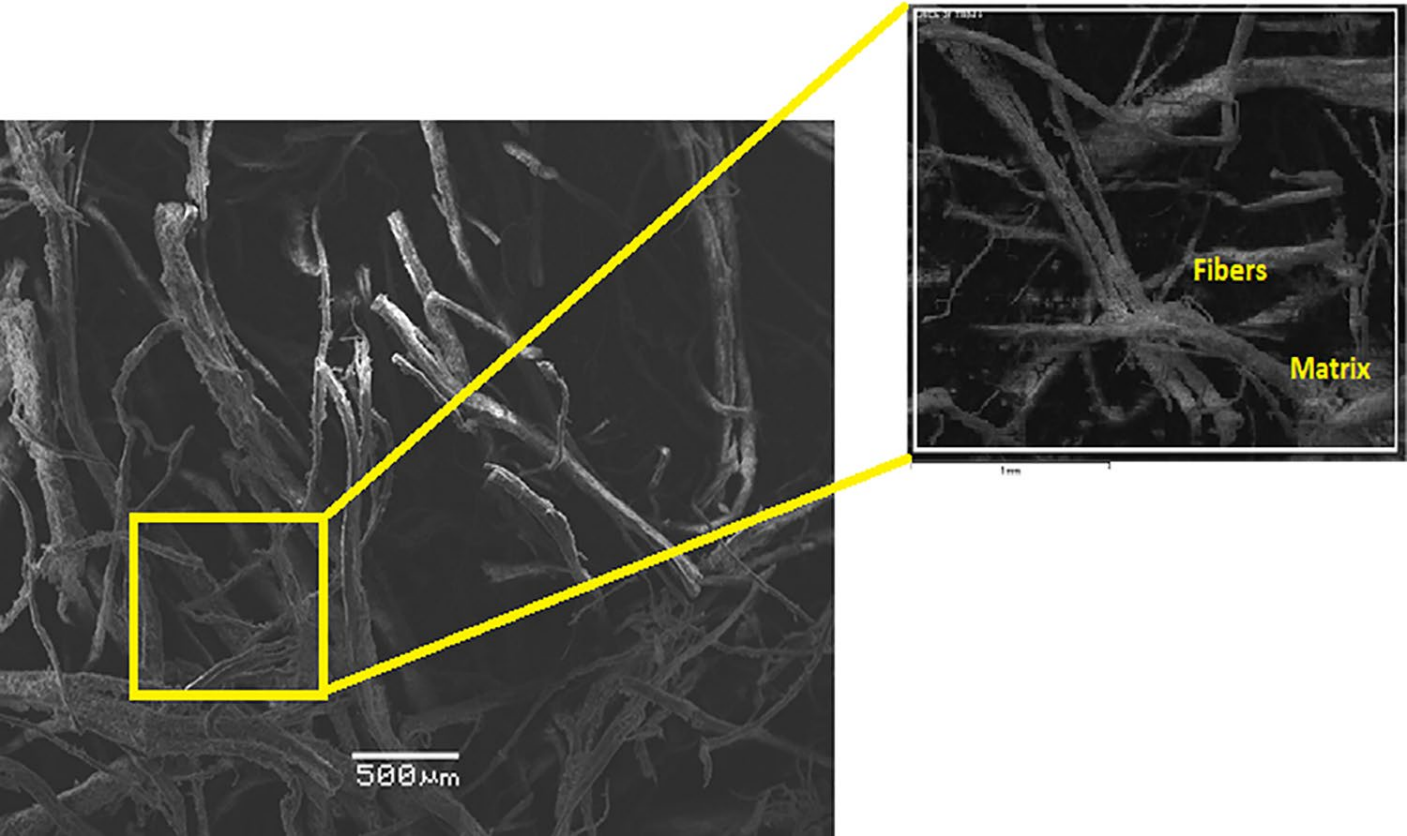
SEM image of hemp limecrete at hemp 2%.

**Figure 10:**
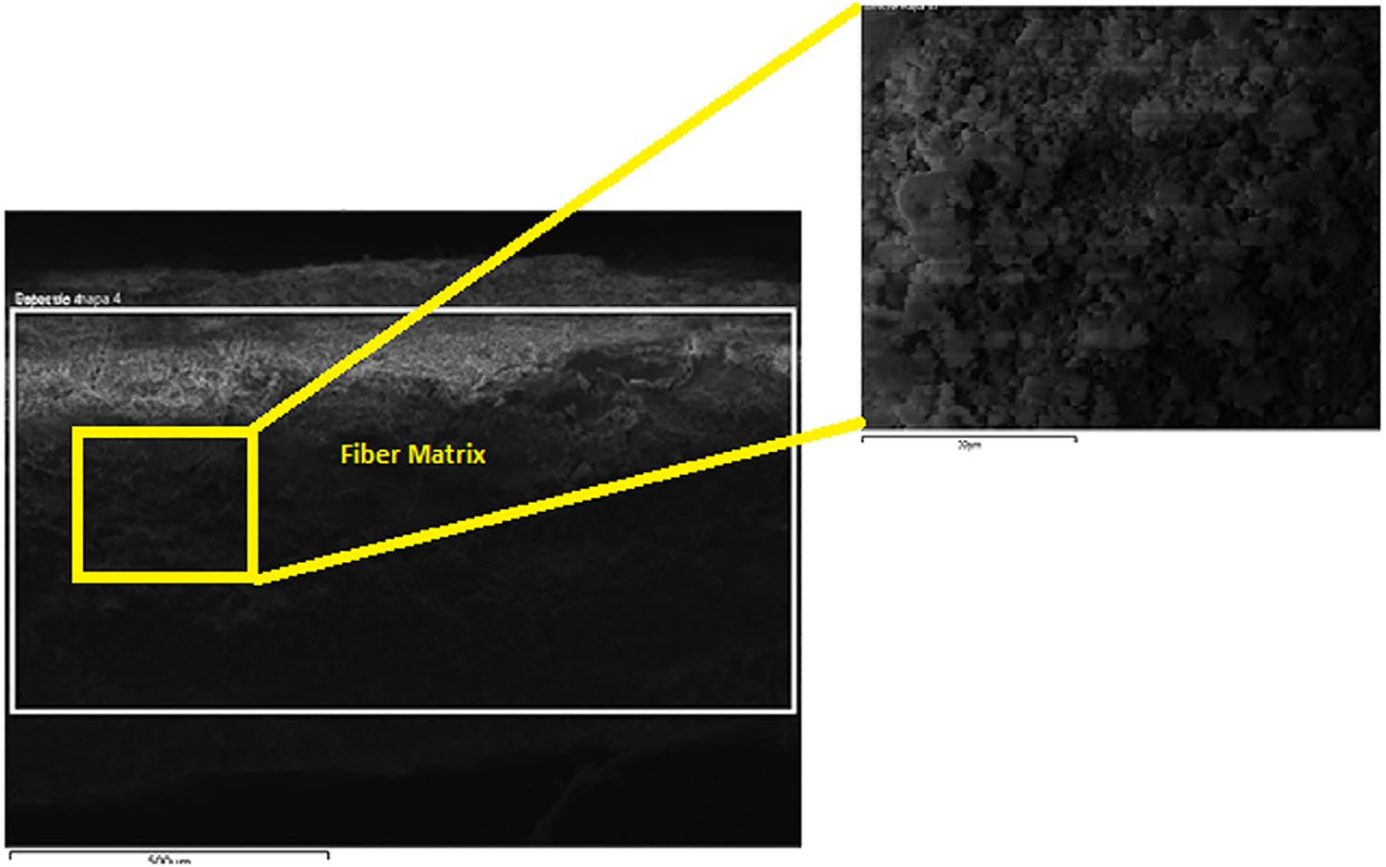
SEM image of hemp limecrete at hemp 4%.

**Figure 11:**
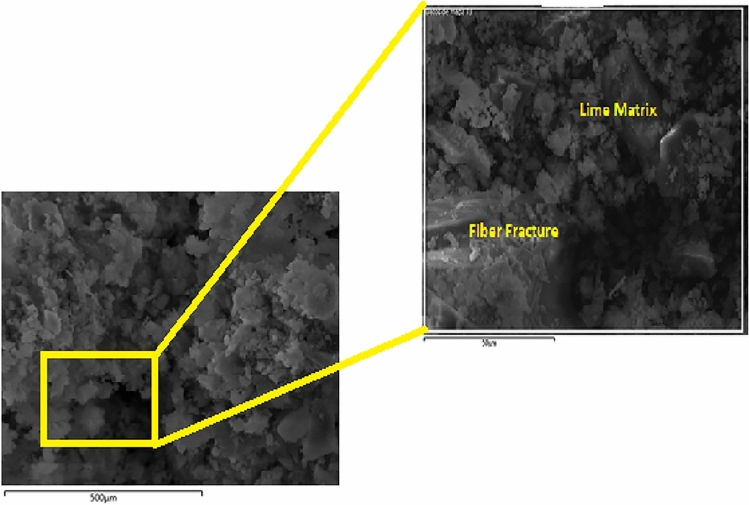
SEM image of hemp limecrete at hemp 6%.

In order to investigate the microstructural characteristics of the specimens, a study using a scanning electron microscope (SEM) was carried out on 2, 4, and 6% hemp mortar with fiber lengths of 0.5, 1.0, and 2.0 mm. The acquired SEM images of lime-based hemp mortar are shown in Figs. 9, 10 and 11. The control specimen which depicts an unreinforced mortar is shown in Fig. 9. It reveals that there are almost no fractures in the limecrete on control Specimen. However, the hemp fiber reinforced limecrete specimen reveals a number of microscopic fractures and cavities. The presence of random fibers in the matrix generates a binding effect, increased toughness, and reduced shrinkage in composite materials, as reported by researchers^42–44^. Therefore, the introduction of hemp fibers into the mortar can lead to an improvement in both the compressive and tensile strengths of the specimens.

In the case of the 2% hemp limecrete, scanning electron microscopy analysis revealed that the hemp fibers were well distributed throughout the matrix, and that the lime binder had penetrated the fibers, creating a strong bond between the two components. The lime was observed to be present as a fine, crystalline structure within the matrix, which also helped to create a strong and durable composite material. The hemp fibers themselves were observed to be relatively uniform in size and shape, with a fibrous texture that allowed them to interlock with one another, providing additional strength to the material.

The addition of 4% hemp to limecrete resulted in a slight increase in the thermal conductivity of the composite compared to the 2% hemp composite. This is likely due to the increased density of the 4% hemp composite, which can lead to better thermal conductivity. Scanning electron microscopy analysis showed that the 4% hemp lime composite had a more homogeneous structure than the 2% hemp composite, likely due to the improved packing of the hemp particles in the mixture. This homogeneity can further improve the overall properties of the composite material. Additionally, the results of the SEM analysis demonstrated that the hemp particles were well distributed within the composite, indicating good adhesion between the hemp and the lime binder. These findings suggest that the addition of 4% hemp can lead to improved thermal conductivity and overall structural properties of hemp limecrete.

In the case of the 6% hemp limecrete, the reduction in compressive strength may be attributed to the higher percentage of hemp. Although hemp fibers can improve the durability and ductility of the material, too high of a concentration can negatively impact the strength. This may be due to the fact that hemp fibers are less stiff and strong than the lime binder, leading to weaker bonding between the fibers and matrix. Additionally, the higher concentration of hemp may have caused a less uniform distribution of fibers throughout the mortar, leading to weaker regions with a lower concentration of fibers. Overall, it is important to find a balance between the benefits of adding hemp fibers and the potential reduction in strength that may occur with higher concentrations.

The SEM analysis of the hemp mortar specimens revealed that there were a significant number of microcracks and voids present in the matrix. These microcracks are often the result of defects in the production process or improper handling. However, these cracks are not visible to the human eye. The hemp fibers can also be seen quite clearly in the limecrete, as shown in Fig. 9. The increasing amount of fibers in the matrix can lead the fibers to knock and overlap each other, which ultimately results in a loss of cohesion with the matrix and a weakened composite material, as reported by previous research^45,46^. This circumstance can lead to a decline in the strength of the hemp fiber specimens.

### Energy dispersive spectrometer (EDS)

The energy dispersive spectrometer (EDS) analysis was conducted on three different hemp limecrete specimens containing 2%, 4%, and 6% hemp, respectively. The weight concentration of elements such as oxygen, calcium, silicon, and aluminum were found to be higher in all Hemp limecrete specimens as compared to traditional limecrete. The EDS analysis also identified the presence of calcium silicate hydrate with a Ca/Si ratio of 1.02 and 2.49 in the 4% and 6% hemp limecrete specimens, respectively. The elemental composition is provided in Table 5.

**Table 5:** Elemental concentration in spceimens of hemp lime fiber mortar.

2% hemp		4% hemp		6% hemp	
Element	Atomic %	Element	Atomic %	Element	Atomic %
O (Oxygen)	51.35	O	57.95	O	55.70
Ca (Calcium)	5.13	Ca	7.52	Ca	4.07
C (Carbon)	42.17	C	32.44	C	38.50
Si (silicon)	0.58	Si	1.03	Si	0.82
AI (Aluminum)	0.31	AL	0.36	AL	0.34
K (Potassium)	0.07	K	0.10	K	0.06
Mg (Magnesium)	0.10	Mg	0.14	Mg	0.18
S (Sulfur)	0.29	S	0.46	S	0.32
Total	100.00	–	100.00	–	100.00

**Figure 12:**
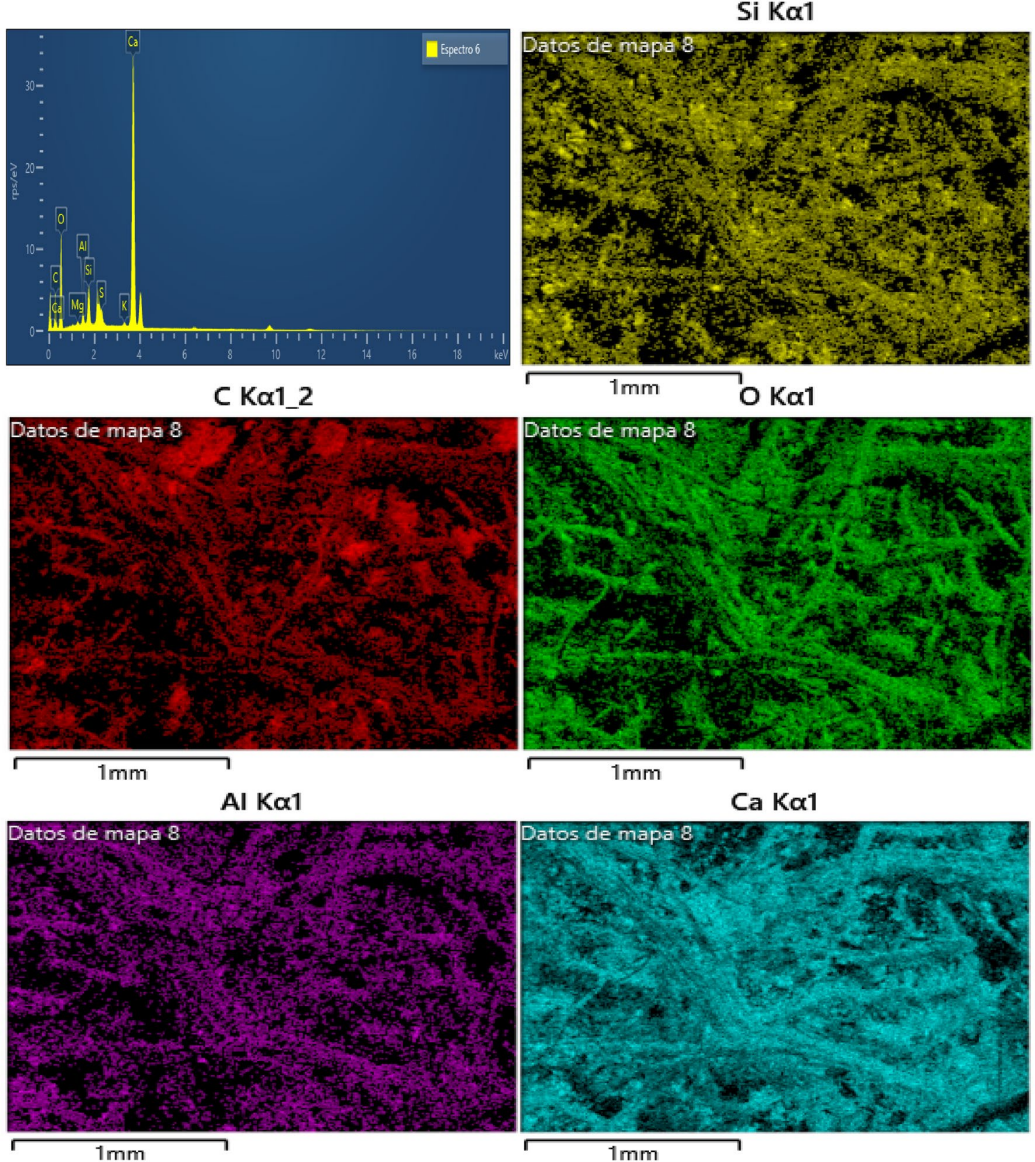
EDS results of hemp limecretes of hemp 2%.

Figure 12 of EDS images shows the weight concentration of elements in the 2% hemp limecrete specimen. Oxygen (O) was the most abundant element, accounting for 51.35% of the total weight, followed by calcium (Ca) at 42.17%. Carbon (C) accounted for 5.13%, while silicon (Si) and aluminum (Al) made up only 0.31% and 0.58%, respectively. Potassium (K), magnesium (Mg), and sulfur (S) were present in trace amounts.

**Figure 13:**
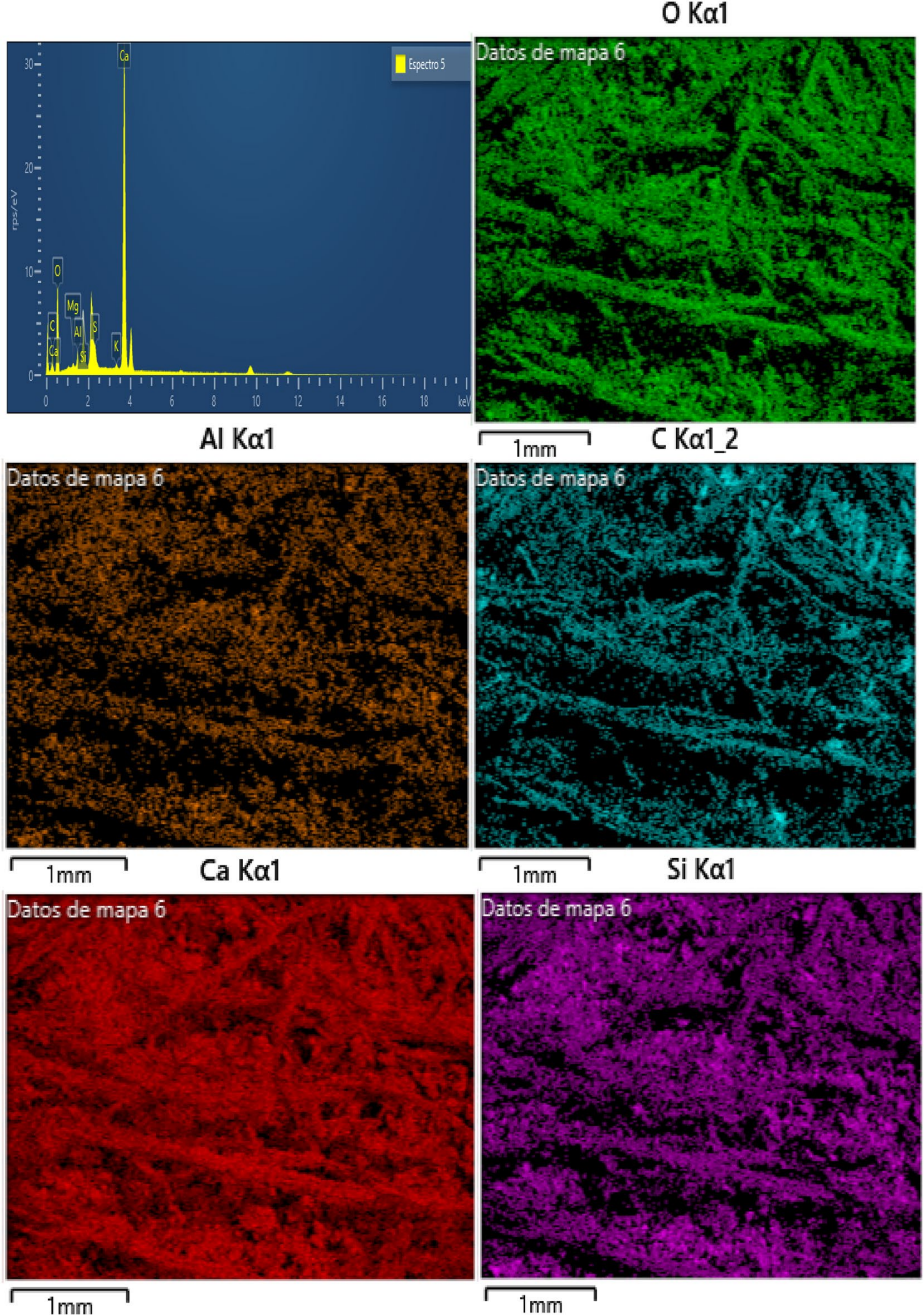
EDS results of hemp limecretes of hemp 4%.

Figure 13 of EDS image shows the weight concentration of elements in the 4% hemp HLM specimen. Oxygen (O) was again the most abundant element, accounting for 55.7% of the total weight, followed by calcium (Ca) at 4.07%. Carbon (C) accounted for 38.5%, while silicon (Si) and aluminum (Al) made up only 0.82% and 0.34%, respectively. Magnesium (Mg), potassium (K), and sulfur (S) were present in trace amounts.

**Figure 14:**
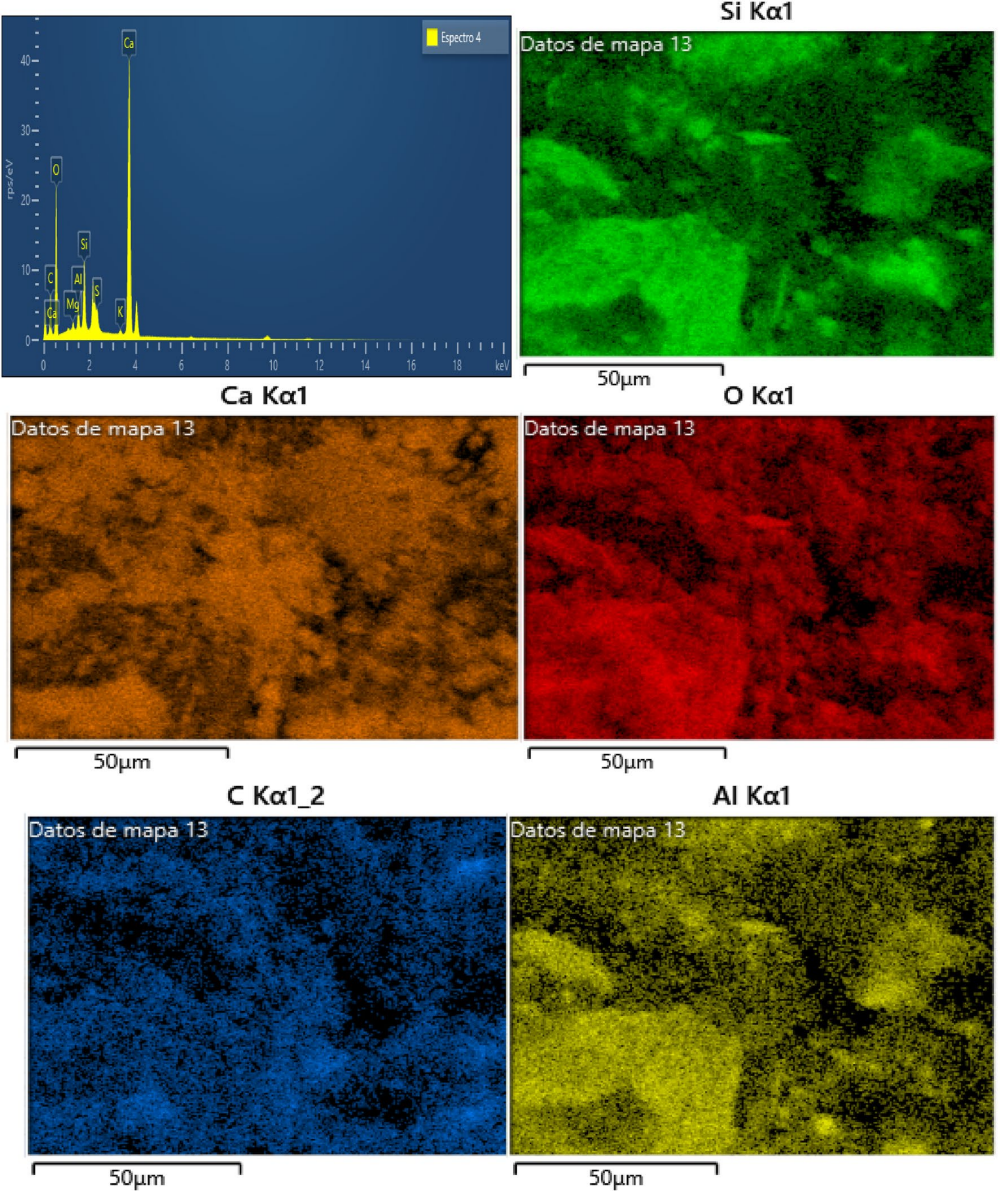
EDS results of hemp limecretes of hemp 6%.

Figure 14. of EDS images shows the weight concentration of elements in the 6% hemp lime mortar specimen. Oxygen (O) was the most abundant element, accounting for 57.95% of the total weight, followed by calcium (Ca) at 7.52%. Carbon (C) accounted for 32.44%, while silicon (Si) and aluminum (Al) made up 1.03% and 0.36%, respectively. Magnesium (Mg), potassium (K), and sulfur (S) were again present in trace amounts. The high presence of elements such as calcium, silicon, and aluminum, in addition to the organic content of the hemp fibers, contributes to the unique mechanical properties of hemp limecrete. The increase in hemp content in the HLM specimens was found to increase the weight concentration of C, Si, and Mg, while decreasing the weight concentration of Ca.

The SEM and EDS analyses of the 2, 4, and 6% hemp mortar specimens reveal the presence of microcracks and voids in the matrix. The introduction of hemp fibers into the mortar leads to an improvement in both the compressive and tensile strengths of the specimens. However, an increasing amount of fibers in the matrix can lead to a decline in the strength of the specimens. The chemical composition analysis of the hemp fiber-limecrete specimens reveals the high presence of elements such as C, Ca, Si, and Al, which improves the bond characteristics of the mortar. These findings can be useful in the development of hemp fiber mortar.

## Conclusion

In conclusion, hemp limecrete has shown promising results in terms of its mechanical and physical properties. The use of hemp shivs as an aggregate in lime-based binder has led to a lightweight and breathable material. Additionally, hemp fibers can be added to the mixture to further enhance its mechanical properties, such as compressive and flexural strength. The optimal fiber length for hemp limecrete has been found to be 1 cm, with an optimum fiber ratio of 4%.

Using 4% fiber increased the Bulk Density values of the mixtures, but Bulk Density values decreased with 6% fiber depending on processability. As fiber length and ratio increased, porosity values of mixtures also increased.

At 7-day test, flexural strength decreased as fiber ratio increased, but increased relatively with increased fiber length. In 28-day mixtures, flexural strength increased as fiber ratio increased, indicating an improvement in the fiber-matrix interface with improved hydration.

Compressive strength decreased as fiber ratio increased in 7 and 28-day mixtures, but using 1 cm long fibers can increase compressive strength. With 2% hemp fiber, compressive strengths of 3.48 MPa is obtained.

The best performance in terms of fiber length is determined to be 1 cm, and the optimum fiber ratio for flexural strength is 4%. Overall, using fibers in mortars can have both positive and negative effects on different properties, and the optimal fiber length and ratio depend on the specific property being considered.

The chemical composition analysis of the hemp fiber-lime- mortar specimens from EDS reveals the high presence of elements such as C, Ca, Si, and Al, which improves the bond characteristics of the mortar.

In conclusion, hemp limecrete has shown to be a promising and sustainable building material with various benefits. The addition of hemp fibers can improve the mechanical properties of the mortar. However, further research is needed to fully understand the long-term durability and behavior of hemp limecrete under various environmental conditions. Overall, the use of hemp limecrete presents an opportunity to create more sustainable and eco-friendly buildings.

## Data Availability

All data generated or analyzed during this study are included in this published article.
